# The prevalence in the general population of advance directives on euthanasia and discussion of end-of-life wishes: a nationwide survey

**DOI:** 10.1186/s12904-015-0068-1

**Published:** 2015-12-07

**Authors:** Aline De Vleminck, Koen Pardon, Dirk Houttekier, Lieve Van den Block, Robert Vander Stichele, Luc Deliens

**Affiliations:** End-of-Life Care Research group, Ghent University & Vrije Universiteit Brussel (VUB), Laarbeeklaan 103, 1090 Jette, Belgium; Department of Family Medicine and Chronic Care, Vrije Universiteit Brussel (VUB), Brussels, Belgium; Heymans Institute of Pharmacology, Ghent University, Ghent, Belgium; Department of Medical Oncology, Ghent University Hospital, Ghent, Belgium

**Keywords:** Advance care planning, Advance directive, End-of-life care wishes, Cross-sectional, General population

## Abstract

**Background:**

To determine the extent to which members of the general population have talked to their physician about their wishes regarding medical treatment at the end of life, to describe the prevalence of advance directives on euthanasia, and to identify associated factors.

**Method:**

This study used data from the cross-sectional Health Interview Study (HIS) 2008 that collected data from a representative sample (*N* = 9651) of the Belgian population.

**Results:**

Of all respondents, 4.4 % had spoken to their physician about their wishes regarding medical treatment at the end of life, while 1.8 % had an advance directive on euthanasia. Factors positively associated with discussions regarding wishes for medical treatment at the end of life were being female, being older in age, having poorer health status and having more GP contacts. People older than 55 years and living in Flanders or Brussels were more likely than the youngest age categories to have an advance directive on euthanasia.

**Conclusion:**

Younger people, men, people living in the Walloon region of Belgium, people without a longstanding illness, chronic condition or disability and people with few GP contacts could represent a target group for education regarding advance care planning. Public information campaigns and education of physicians may help to enable the public and physicians to engage more in advance care planning.

## Background

Advance care planning (ACP) has gained international attention for its perceived benefits in enhancing patient autonomy, ensuring better quality of care and improving quality of life in the final stages of life [1, 2]. ACP is the process through which patients are able to express their preferences regarding end-of-life care [3, 4]. ACP involves discussions about goals of care and preferences for treatment between patients and health professionals, which may involve family members or friends. ACP may include the designation of a surrogate decision-maker to make future health care decisions for the patient, or the completion of an advance directive (AD) [5].

A recently published systematic review showed that interventions including communication about ACP improved the quality of communication and concordance between patient preferences and end-of-life care received [2]. Because of these benefits, ACP is seen as a useful behaviour to promote among the general public and in several countries public health campaigns to encourage it have been put in place such as the Speak Up campaign in Canada or the Dying Matters initiative in the UK [6, 7]. Nonetheless, little information is available on the involvement of the general public in ACP and the extent to which people actually plan their end-of-life care, which is the focus of this population-based study of the Belgian general population. Improved understanding of the public’s involvement in ACP could help inform the development of public policy. Determining which subgroups of the population are engaged in ACP and which are not can help to define the communication and health campaigns about ACP within a context that is meaningful to the public [8].

In Belgium the Law on Patients’ Rights (2002) gives people the right to reject any medical treatment and to appoint a surrogate decision-maker to advocate for their rights if they are unable to make decisions or speak for themselves [9]. Refusal of treatment can be documented in a legally binding negative AD, also known as a living will, which is similar to those in the Netherlands, the USA and Canada. Furthermore, people can document in advance specific wishes for end-of-life care in an advance statement, also called a positive AD, which is indicative but not legally binding on the physician. These advance statements are called “positive” ADs because they are about what patients would still want if they could no longer ask for themselves, as opposed to negative ADs to refuse treatments or examinations. Belgium is a specific case as it recognizes a type of positive AD that does not exist in most other countries. In 2002, the Belgian Parliament legalised euthanasia, i.e. the use of life-ending drugs by a physician on explicit patient request. People in Belgium can draft an AD on euthanasia in case they find themselves in specific situations of lack of capacity [10, 11]. The law on euthanasia allows people with mental capacity to draw up a prior declaration of intent to request euthanasia should they be in an irreversible state of unconsciousness and no longer able to ask for euthanasia themselves. In practice, this means that an AD on euthanasia only applies to those in an irreversible coma. A request for euthanasia is not legally binding and acts as a guide for the treating physician. ADs on euthanasia may be registered at the city hall, but this is not mandatory. In 2013, a total of 20,414 people in Belgium registered an AD on euthanasia, an increase compared with 12,728 people in 2012.

The aim of this study is firstly to determine to what extent members of the general population have talked to a physician about their wishes regarding medical treatments at the end of life and what the prevalence is of an AD on euthanasia in the general population and secondly to determine to what extent socio-demographic characteristics, health status and health service use are associated with the involvement in ACP.

## Methods

### Design and population

This study uses data from the cross-sectional Health Interview Study (HIS) that collects data from a large representative sample of the Belgian population. The HIS is organized by the Belgian Scientific Institute of Public Health (WIV-ISP) and was conducted for the fourth time in 2008–2009. Around 6000 private households are randomly selected from the National Population Register using a multistage stratified clustered sampling process. Of each selected household, a maximum of four members are eligible. The householder and partner are always selected, as well as two extra randomly selected members (or three if there is no partner). This study includes only participants older than 15 years.

### Questionnaire

Involvement in ACP was analyzed based on two items: (1) Have you ever spoken to your physician about your wishes regarding medical treatments at the end of life? and (2) Do you have an advance directive requesting euthanasia?. Possible responses were Yes and No. Data collection is performed via a self-administered questionnaire filled in by each selected person aged 15 years or older. However, only respondents aged 18 years or older are included in the analysis, because an advance directive on euthanasia can only be drafted by people older than 18.

Several procedures were used to ensure data quality: the content of the HIS questionnaire was discussed in workgroup sessions with academic experts, health government agencies and fieldwork experts. The questionnaires were pre-tested: firstly, questions from other European surveys that were added to the HIS 2008 questionnaire benefited from a large scale pre-test [12] and secondly, the HIS questionnaire was pre-tested by the WIV-ISP in a small, diverse sample of people to evaluate the length, comprehension, readability etc. of questions.

### Analysis

The selection of the independent variables is based on their possible influence on involvement in ACP or the formulation of ADs, as shown in previous research [13–15]. Socio-demographic measurements include gender, age, educational level, marital status and region of residence. Health-related measurements include health status (having a longstanding illness, chronic condition or disability) and health service utilization (mean number of GP contacts in the past 12 months and mean number of specialist contacts in the past 12 months).

The sample is weighted according to the stratified clustered sampling design of the survey to be representative of the Belgian population. Involvement in ACP is described using frequencies. The independent characteristics were tested for significant association with involvement in ACP using Pearson chi2 test (not shown in paper) and, when found significant, entered in multivariate logistic regression models in order to control for confounding effects and investigate their association with the dependent variables. Confidence intervals are calculated at the 95 % level. Analyses are conducted with SPPS 22.0 software using the complex samples procedure to account for the complex survey design.

### Ethical considerations

The protocol of the Belgian HIS 2008 is approved by the Superior Council of Statistics [12]. Submisson to the Ethical Committee of the WIV-ISP was not needed, because it is a recurrent project that had been approved in 2004. Providing the data by the participants involved giving implied consent (not written).

## Results

The participation rate among the contacted households was 55 % (around 6,000 households were contacted). Of the 9651 respondents, 52.1 % were female, 26.2 % were between the ages of 18 and 34, 37.5 % were between the ages of 35 and 54, 25.6 % between 55 and 74 and 10.7 % 75 years or older (Table 1). For 38.7 % of the respondents the highest educational level within the household was higher education (post-secondary). More than half of the respondents were married or had a registered civil relationship (56.6 %) and lived in Flanders (58.3 %). Of all respondents 32.7 % suffered from a longstanding illness, chronic condition or disability. Around a quarter had seen their GP once every two months in the last year and the majority had not seen a specialist in the past 12 months (77.5 %).

**Table 1:** Participant characteristics of the sample of the Belgian general population (*N* = 9651) and proportions having spoken about wishes regarding medical treatments at the end of life with their physician and having an AD on euthanasia

		Unweighted sample (Weighted %)	Has spoken about wishes regarding medical treatment at the EOL to their physician	Has an AD on euthanasia
		*N* (%)	%	%
Total respondents			4.4	1.8
Socio-demographic factors				
Gender	Man	4244 (47.9)	3.3*	1.7
	Woman	5080 (52.1)	5.4*	1.8
Age	18-34	2222 (26.2)	0.7*	0.5*
	35-54	2906 (37.5)	3.5*	0.9*
	55-74	2216 (25.6)	6.8*	3.3*
	75+	1980 (10.7)	12.0*	4.6*
Highest educational level within the household	≤ Primary education	1437 (12.2)	6.7*	2.7
	Lower secondary	1511 (15.9)	5.9*	1.8
	Higher secondary	2770 (33.2)	4.8*	2.0
	Higher education	3302 (38.7)	2.9*	1.4
Marital status	Single (never married)	2399 (26.4)	2.0*	1.1*
	Married or registered civil relationship	4672 (56.6)	3.9*	1.7*
	Widow/er (not remarried)	1428 (8.2)	13.0*	4.5*
	Divorced (not remarried)	804 (8.7)	7.3*	1.7*
Region of residence	Flemish region	3304 (58.3)	3.8*	2.0*
	Brussels’ region	2750 (10.6)	4.8*	2.5*
	Walloon region	3270 (31.1)	5.5*	1.1*
Health status				
Longstanding illness, chronic condition or disability	Yes	3437 (32.7)	7.8*	2.6*
	No	5857 (67.3)	2.7*	1.4*
Health service use				
Number of GP contacts	Never	4502 (53.2)	2.1*	1.1*
	1×/2 months	2447 (26.9)	5.2*	2.0*
	1×/month	1412 (12.5)	8.5*	3.2*
	≥1×/month	811 (7.4)	11.8*	3.0*
Number of specialist contacts	Never	6930 (77.5)	3.6*	1.6
	1×/2 months	1384 (14.4)	6.3*	1.8
	1×/month	496 (5.1)	7.3*	3.9
	≥1×/month	302 (3.0)	10.6*	2.4
Involvement in ACP				
Has spoken about wishes regarding medical treatments at the EOL to their physician		412 (4.4)	-	22.2*
Has an AD on euthanasia		167 (1.8)	55.4*	-

Abbreviations: *GP* general practitioner, *EOL* end of life

Sums may not always amount to the total sample number because of missing values on variables. Percentages may not always add up to 100 because of rounding. Percentages are row percentages

Missing values: for gender *n* = 0, age *n* = 0, for highest educational level in the household *n* = 304 (3.3 %); for marital status *n* = 21 (0.2 %); for region of residence *n* = 0; for having a longstanding illness, chronic condition or handicap *n* = 30 (0.3 %); number of GP contacts *n* = 152 (1.6 %); number of SP contacts *n* = 212 (2.3 %); for having spoken about wishes regarding medical treatments at the EOL to their physician *n* = 2109 (22.6 %); for having an AD on euthanasia *n* = 2128 (22.8 %)

* Significant at *p* <0.05 using Pearsons Chi2 test

### Involvement in advance care planning

Of all respondents, 4.4 % indicated they had talked to a physician regarding medical treatments at the end of life and 1.8 % said they had an AD on euthanasia (Table 1). Of all respondents who had discussed their wishes regarding medical treatment at the end of life with a physician, 22.2 % had an AD on euthanasia. Vice versa, 55.4 % of all respondents who had an AD on euthanasia, had discussed their wishes regarding medical treatment at the end of life with a physician. Women, older persons, the widowed and people who suffer from a longstanding illness, chronic condition or disability had spoken more often about their wishes regarding medical treatment at the end of life, as had those who saw a GP or specialist more than once a month. Having an AD on euthanasia was also more common among older respondents, the widowed and people with a longstanding illness, chronic condition or disability. Remarkably, people with a lower educational level had spoken more with a physician about medical treatments at the end of life and had more often an AD on euthansia than people with a higher educational level. However, a crosstabulation between age and educational level showed that the majority of older people were represented in the lowest educational levels, while the majority of younger had a higher educational level (not shown in the paper).

### Factors associated with involvement in advance care planning

The probability of having spoken to a physician about wishes regarding medical treatments at the end of life was higher for women (OR = 1.5) and those with a longstanding illness, chronic condition or disability (OR = 1.5) (Table 2). The probability of having discussed their wishes with a physician also significantly increased with age and with the number of GP contacts.

**Table 2:** Factors associated with involvement in ACP of a representative sample of the Belgian general population

		OR (95 % CI) for yes *vs*. no*	
		Spoken about wishes regarding medical treatments at EOL to physician	Having an advance directive requesting euthanasia
Socio-demographic characteristics			
Gender	Man (ref)	Ref	
	Woman	**1.5 [1.1–2.0]**	
Age	18-34 (ref)	Ref	Ref
	35-54	**5.1 [2.6–9.9]**	2.1 [0.8–5.1]
	55-74	**8.2 [4.1–16.4]**	**6.4 [2.7–14.9]**
	75+	**11.1 [5.2–24.0]**	**6.3 [2.1–18.4]**
Region of residence	Flemish region (ref)		Ref
	Brussels’ region		1.5 [0.9–2.4]
	Walloon region		**0.5 [0.3–0.9]**
Health status			
Having a longstanding illness, chronic condition or handicap	Yes	**1.5 [1.1–2.0]**	
	No (ref)	Ref	
Health care use			
Number of GP contacts	Never (ref)	Ref	
	1×/2 months	**1.7 [1.1–2.6]**	
	1×/month	**2.2 [1.3–3.5]**	
	≥1×/month	**3.0 [1.7–5.2]**	

Abbreviations: *OR* odds ratio, *ref* reference category, *Ns* not significant

*Odds Ratio with 95 % confidence interval from complex multivariate logistic regression analysis

Bold denotes significant at *p* < .05

Compared with the youngest age category, people older than 55 years were more likely to have an AD on euthanasia. Those living in the Walloon region of Belgium were less likely to have an AD on euthanasia compared with those living in Flanders (OR = 0.5).

## Discussion

This study shows that 4.4 % of a representative sample of the Belgian general public have spoken about their wishes regarding medical treatments at the end of life, while 1.8 % have an AD on euthanasia. Discussions with a physician regarding wishes for medical treatment at the end of life were more likely to have taken place among women, as people get older, among people with a poorer health status and those having more GP contacts. Having an AD on euthanasia was more likely for people older than 55 years and living in Flanders or Brussels.

Discussions regarding wishes for medical treatment at the end of life with physicians are relatively rare among the Belgian general public. Even among those who have an AD on euthanasia only half of respondents (55.4 %) had discussed their wishes regarding medical treatment with a physician. However, an AD on euthanasia does not need to be discussed with a physician, something about which some people might be hesitant. An AD on euthanasia must be drafted in the presence of two adult witnesses and they are responsible for notifying the treating physician of its existence should the patient fall into an irreversible coma. People can choose either to deliver copies of their AD on euthanasia to a number of people (of whom their physician might be one) or to register it at the city hall in a federal database, but this is not mandatory. It is however also possible that people do visit their physician with the intention of discussing the completion of an AD on euthanasia, but that the physician omits to take up the opportunity to elaborate on their wishes for medical treatment at the end of life.

A cross-sectional survey in the Netherlands (one of the three countries, with Belgium and Luxembourg, where euthanasia has been legal since 2002) showed that 13 % of the general population had discussed issues related to medical decision-making at the end of life with a physician [13]. It is known that, especially in the Netherlands, patients prioritize autonomy and control during the dying process [16]. Cross-country studies also repeatedly found that Dutch physicians discuss end-of-life issues more frequently than their European counterparts [17–19]. Of the Dutch general population, 3 % reported they have an AD on euthanasia compared with 1.8 % in the present study. Internationally, the interest of people in making ADs refusing medical treatments has been shown to be low, ranging between 18 % and 34 % in the general population of the USA [20–23] and between 3 % and 19 % in the general population in Europe [14, 24].

A number of characteristics associated with the public’s engagement in ACP are consistent with earlier studies. Women and those with a serious illness or increased dependency have been shown to discuss their end-of-life care preferences more often with physicians or have higher AD completion rates [13, 14, 20, 25]. As may be expected, older people were also more likely to have discussed or documented their end-of-life care wishes than those in the youngest age categories [13, 26]. On the one hand, evidence suggests that people in general are unwilling to engage in ACP until they grow older or become ill; a lack of information, procrastination or avoidance could be important reasons for the low completion rates among younger people [27, 28]. Younger people in good health tend not to feel the need for ACP. On the other hand, physicians are also hesitant to initiate these discussions and often believe that ACP is unnecessary for young and healthy patients which compounds these barriers [29, 30]. And although those who have been widowed are more involved in the process of ACP, marital status was surprisingly not a predictor of engagement in ACP in this study [31]. A population-based study on AD completion in Alberta showed that people who had looked after or given care to a dying person were more likely to complete an AD [32]. The authors argue that experience of death and dying are likely to have a greater impact on having an AD than socio-demographic characteristics. In our study, older age was a notable predictor of AD completion, but experiences with death and dying were not asked about. Older people, who are in general more likely to be widowed, could have experienced a death in their close environment. Also, the specific type of AD examined in this survey was an AD on euthanasia as opposed to an AD for medical treatment. Possibly, lived experiences could also greatly influence the level of involvement in ACP in Belgium. This is a focus for future research.

Remarkably, people living in the Walloon region of the country were less likely to have an AD on euthanasia compared to people living in the Flemish part of Belgium. Unfortunately, reasons for the identified differences between the regions in Belgium could not be explored. Previous research on end-of-life care in Belgium has suggested a difference in medical culture between the Dutch-speaking and French-speaking community, with a stronger appreciation of curative, technological and specialist medicine in the French-speaking community [33]. Perhaps, societal or culturally determined attitudes towards euthanasia might also differ between the regions in Belgium and influence the prevalence of an AD on euthanasia. However, these hypotheses need more research before solid conclusions can be drawn. This study also shows that having more contact with a GP was associated significantly with discussion of end-of-life care wishes. In the Belgian health care system a strong emphasis is put on primary care and most people have a long-lasting relationship with their GP whom they consult regularly (78 % at least once a year) [34]. Moreover, the number of GP contacts increases exponentially with age (and probably health-related problems) and persons aged 75 or over are seen by their GP on an almost monthly basis. Future interventions might focus on stimulating GPs to initiate ACP discussions in good time as a sudden or serious chronic illness can render any adult incapable of decision-making. The aim of interventions should not only be to encourage the formulation of ADs on euthanasia, as such ADs only apply to very specific medical circumstances as described in the introduction, but to provide adequate information about the different types of ADs to those who are interested and to make the process of completing an AD an opportunity to have important conversations with physicians, family and friends.

Even though a previous study has shown that the majority of people in Belgium are open to discussions on end-of-life care [35], only a small percentage of the population had discussed their wishes regarding medical treatment at the end of life with a physician. This suggests that the stimulation of both patients and physicians to engage in end-of-life care discussions would be useful to enhance ACP in practice. Public information campaigns can increase awareness among the Belgian general public regarding the importance of timely ACP discussions. This study shows that younger people, men, those living in the Walloon region of Belgium and those with few GP contacts are a target group for education. Nonetheless, older people represent another key target group, as they are at higher risk of needing end-of-life care [32]. Of those older than 75 years, only 12 % had ever had a discussion with their physician about their wishes regarding medical treatment at the end of life. And although guidelines suggest that ACP should be initiated with people who suffer from a chronic, life-limiting illness, our results show that only 7.8 % of people with a poorer health status had ever spoken with a physician about their wishes for medical treatment at the end of life. Public information campaigns can help to overcome important barriers to engagement in ACP, including the perception that ACP is irrelevant or the possession of insufficient information to engage in such discussions [5, 28]. Secondly, a more active role for the physician in initiating such discussions could also enhance ACP. It has been suggested before that physicians have the responsibility to inform their patients and to initiate discussions in a timely manner [4, 36, 37]. However, they need to be trained and supported in how to do this, they often delay communication until the end of life or wait for patients to raise the topic [38–40].

This is the first study on the general public’s involvement in ACP in Belgium. It is a population-based study founded on a representative sample of the Belgian population. Other important strengths include the large sample size, the robustness of the methodology and the quality of research procedures. We used data from the HIS, which has a long history of data collection in the Belgian population and is not based only on a specific interest in end-of-life care. However, this study also has some limitations. Firstly, the specific context of Belgium as one of the three countries where euthanasia is legal, might hamper the generalizability of our results to other settings. Secondly, because of the low response-rate (55 %), non-response bias cannot be excluded. The missing values for the outcome variables are around 23 % and non-response analysis showed that missing values were more likely to be male, older, have a lower educational level and live in Wallonia or Brussels. As a result, it is possible that some of our findings are biased because of non-response. Thirdly, because this study examined the respondent’s own report of their involvement in ACP, the results may be subject to recall bias.

## Conclusion

Few people in Belgium have discussed their wishes regarding medical treatment at the end of life with their physician or have completed an AD on euthanasia. Younger people, men, people living in the Walloon region of Belgium, people without a longstanding illness, chronic condition or disability and people with few GP contacts might represent a target group for education as they are less likely to engage in ACP. Public information campaigns and the education of physicians may encourage the public to engage in ACP and help to enable patients, families and physicians to have more conversations about care at the end of life.

## Abbreviations

ACP: advance care planning
AD: advance directive
GP: general practitioner
